# Parental Adherence to Ideal Cardiovascular Health Status Was Associated With a Substantially Lower Prevalence of Overweight and Obesity in Their Offspring Aged 6–18 Years

**DOI:** 10.3389/fnut.2021.715171

**Published:** 2021-09-20

**Authors:** Yanhui Li, Di Gao, Zhaogeng Yang, Ying Ma, Manman Chen, Jun Ma, Yanhui Dong, Bin Dong

**Affiliations:** Institute of Child and Adolescent Health, School of Public Health, Peking University, Beijing, China

**Keywords:** cardiovascular health, parental lifestyle and behavior, overweight and obesity, offspring, intergeneration

## Abstract

**Background:** Parental health status had a potential influence on offspring health. This study aimed to investigate the separate associations between paternal and maternal cardiovascular health statuses and the prevalence of childhood overweight and obesity in the offspring.

**Methods:** Data were from a cross-sectional study conducted in seven provinces or cities of China in 2013. A total of 29,317 children aged 6–18 years old and their parents, making up 9,585 father-offspring pairs and 19,732 mother-offspring pairs, were included in the final analysis. Information on parental cardiovascular health status factors (dietary behaviors, body mass index (BMI), smoking, physical activity, hypertension, and diabetes mellitus) was obtained from the structured self-administrated questionnaires. Based on the health status factors, we then generated an ideal cardiovascular health (iCVH) score. The overweight and obesity of children were defined using age- and sex-specific cutoffs based on the International Obesity Task Force criteria. A multilevel log-binomial regression model was used to assess the association between parental cardiovascular health status and prevalence of childhood overweight and obesity in the offspring.

**Results:** The prevalence of pediatric overweight and obesity was 22.0% in the father-offspring subset and 23.8% in the mother-offspring subset, respectively. Fathers with ideal BMI, non-smoking, and absence of hypertension and diabetes, and mothers with ideal BMI, ideal physical activity, and absence of hypertension and diabetes were found to be associated with lower prevalence of overweight and obesity in the offspring. The prevalence of offspring overweight and obesity was significantly decreased with the parental iCVH scores increased. Each additional increase in paternal and maternal iCVH factor was associated with a 30% and 27% lower prevalence of overweight and obesity in the offspring. Compared with children whose parental iCVH scores ≤ 3, offspring whose fathers or mothers met all six iCVH factors had 67% [prevalence ratio (PR): 0.33, 95%CI: 0.25–0.42] and 58% (PR: 0.42, 95%CI: 0.29–0.62) lower prevalence of overweight and obesity, respectively.

**Conclusions:** Parental adherence to iCVH status was associated with a lower prevalence of pediatric overweight and obesity in offspring. Our findings support the intervention strategy that parents should involve in the obesity intervention program for children.

## Introduction

Overweight and obesity in children and adolescents have become an increasingly serious public health concern worldwide. Overall, the prevalence of pediatric obesity increased more than 5-fold for both boys and girls aged 5–19 years from 1975 to 2016, and the rise in body mass index (BMI) continued to accelerate in the East and South Asia (1, 2). Children from China have also experienced a substantial increase in the prevalence of overweight and obesity over the past three decades, with an increase from 3.0% in 1985 (3) to 19.4% in 2014 (4). It has been widely recognized that being overweight or obese in childhood could lead to a series of adverse health consequences throughout the life course, namely, adult obesity, diabetes, cardiovascular disease, and even premature death (1, 5, 6). It is important to know the modifiable factors to prevent overweight and obesity.

Pediatric overweight and obesity are influenced by multiple factors, among them parental health status has been regarded as one of the most important ones. Numerous evidence had suggested that parental cardiovascular-related diseases, such as obesity (7, 8), hypertension (9), and diabetes mellitus (10), were associated with a higher risk of obesity in their offspring. Besides, it has also been shown that parental lifestyle behaviors, namely, smoking, alcohol drinking, physical activity, and diet, could influence the development of obesity in the next generation (11–13). A recent study found that mothers who were physically active, adhered to a healthy diet, maintained a healthy BMI, did not smoking, and had light to moderate alcohol consumption were significantly associated with reduced risk of offspring obesity (12). Although the association of individual parental health-related factors with offspring overweight and obesity has been widely investigated previously, few have assessed the effect of overall parental cardiovascular health status on pediatric overweight and obesity in their offspring. Previous studies have shown that the intergenerational association of the same sex seemed to be stronger due to the role of genetics and epigenetics (14); however, it is unclear whether paternal and maternal health statuses have different degrees of influences on offspring obesity risk.

Based on intergenerational transmission and stronger beneficial effects in overall cardiovascular health factors, we aim to investigate the associations of parental individual and combined cardiovascular health factors, namely, ideal dietary behavior, ideal BMI status, non-smoking, regular moderate to vigorous physical activity, absence of hypertension, and absence of diabetes mellitus, with the prevalence ratio (PR) of childhood overweight and obesity in their offspring, and to analyze the influence of paternal and maternal cardiovascular health on overweight and obesity in boys and girls. Understanding the role of fathers and mothers could help to develop effective intervention strategies.

## Methods

### Study Setting and Population

**Figure 1:**
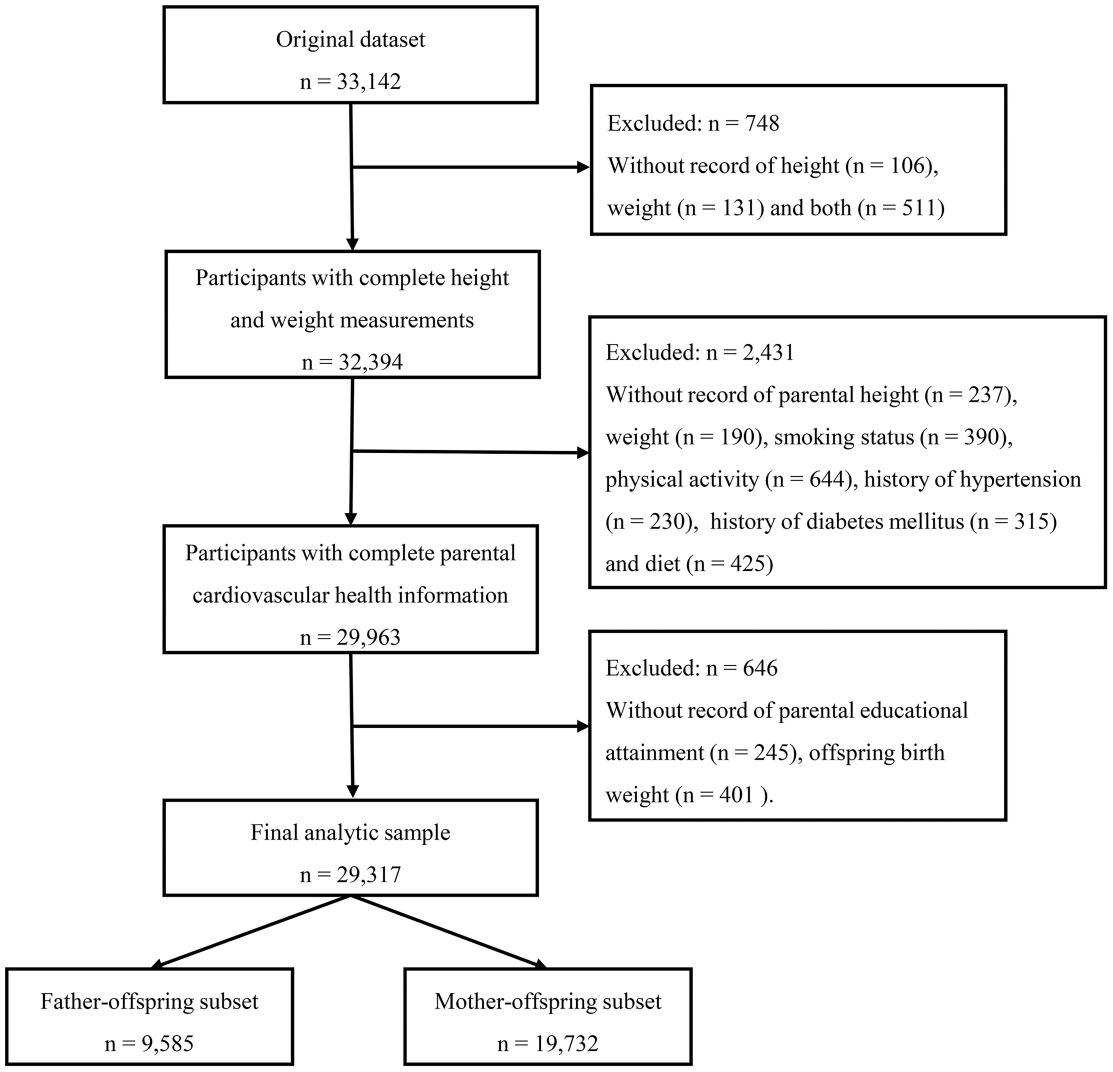
The selection process and flow chart of study participants.

Data in this study came from the baseline survey of a national multicentered cluster randomized controlled trial aimed to reduce the burden of obesity in school-aged children. The survey was conducted in seven provinces or cities of China (Hunan, Ningxia, Tianjin, Chongqing, Liaoning, Shanghai, and Guangzhou) in 2013, more detailed description of the study can be assessed elsewhere (15). Briefly, a multistage cluster sampling method was used to determine the participants. At first, several regions were randomly selected from each province or city, and 12–16 schools were randomly chosen from each region. At each school, two classes were randomly selected in each grade and the whole class students were invited to participate in this survey, then those who signed the informed consent were finally enrolled. All survey sites used the same protocol during the implementation process, and all processes or randomization was performed by a staff member who did not involve in the survey. The selection process and flow chart are displayed in Figure 1, inclusion criteria include those participants who had complete records of height, weight, and parental cardiovascular health status. The final study included 29,317 children aged 6–18 years old, constituting 9,585 father-offspring pairs and 19,732 mother-offspring pairs. This study has been approved by the Ethical Committee of Peking University (IRB No. 0000105213034).

### Data Collection

All enrolled children were asked to undergo an anthropometric measurement, their parents were invited to complete a self-administrated questionnaire, as only one parent (either father or mother) completed the questionnaire, so we only collect the cardiovascular health information of one parent for each child.

Child anthropometric measurements were conducted by trained investigators, according to standard procedure. During the process, participants were asked to wear light clothes and without shoes. Height was measured to the nearest 0.1 cm using a portable stadiometer, and weight was recorded to the nearest 0.1 kg with a scale. Every participant was measured two times and the readings of height and weight were recorded each time, we use the average of the two measurements in data analysis. BMI was calculated as the weight (kg) divided by the square of the height (m^2^), nutritional status of offspring was defined using age- and sex-specific cutoffs based on the International Obesity Task Force criteria (16). The information about child sex, dietary behaviors, and physical activity were collected through children's questionnaires. Children were asked to report the frequency and amount they ate fruits, vegetables, meat and its products, and sugar-sweetened beverages (SSBs) and the frequency and average hours of physical activity over the past 7 days. Children above the fourth grade would fill in a questionnaire of children by themselves in class, instructed by the class teacher, while children of grades 1–3 were reported by parents.

The parental questionnaire was performed to collect information about parental cardiovascular health status. Dietary behaviors consisted of four food items, namely, fruits, vegetables, meat and its products, and SSBs, parents reported the frequency and amount they ate over the past 7 days. The parental BMI was calculated with height and weight data reported in the questionnaire; current smoking status was obtained by asking “whether you have smoked over the past 7 days?”; to obtain information about leisure-time physical activity, the International Physical Activity Questionnaire-Short Form (17, 18) was applied and parents were asked to report the days and average hours in attending moderate- to vigorous-intensity physical activity over the past 7 days; history of hypertension was obtained by asking “whether you have ever been diagnosed with hypertension by doctors or taken drugs to control blood pressure”; history of diabetes mellitus was obtained by asking “whether you have been diagnosed with diabetes mellitus by doctors or taken drugs to control blood glucose”; besides, parents also reported their educational attainment from the following options: (1) junior high school or below, (2) senior high school, (3) junior college, and (4) college or above. The residence area, birth weight, and delivery mode of the children were also collected through the questionnaire.

### Evaluation of Parental Cardiovascular Health Status

We adopted the definition of Life's Simple 7 of the American Heart Association (AHA) (19) as criteria to assess parental cardiovascular health status. Because we did not collect data on parental total serum cholesterol, we remove it and generate six-factor cardiovascular health status scores for parents. In this study, parental cardiovascular health factors included dietary behaviors, BMI, current smoking, physical activity, hypertension, and diabetes mellitus. Each of the six factors was categorized as ideal or unideal, the participant received 1 if he/she met the criteria, otherwise obtained 0. The scores for the six factors were summed to create a total ideal cardiovascular health (iCVH) score ranging from 0 to 6. The iCVH status was categorized into four groups according to the continuous iCVH scores (1–3, 4, 5, and 6). The definitions of ideal BMI status, current smoking, and physical activity were consistent with the AHA definition (19). Ideal hypertension and diabetes mellitus were defined as no history of diagnosis (20). A modified ideal dietary behavior based on our questionnaire was defined following the most recent “Dietary Guidelines for Chinese Residents” (21). The definition of parental iCVH is shown in Table 1.

**Table 1:** Definition of ideal cardiovascular health (iCVH) for parents.

**No**.	**Factors**	**Ideal health status definition**
1	Dietary behaviors	Satisfy 3–4 components^*^ •Fruits: ≥3 servings per day •Vegetables: ≥4 servings per day •Meat and its products: ≤3 servings per week •Sugar-sweetened beverages: ≤4 servings per week
2	Body mass index	<25 kg/m^2^
3	Current smoking	Never
4	Physical activity	≥150 min/week moderate intensity or ≥75 min/week vigorous intensity or combination
5	Hypertension	Absence of hypertension
6	Diabetes mellitus	Absence of diabetes mellitus

* *One serving of fruit or vegetable is ~120 g. One serving of sugar-sweetened beverage is about 250 ml. One serving of meat is ~100 g. The definition was adapted from the recommendations of the AHA and modified according to the Dietary Guidelines for Chinese Residents*.

### Statistical Analyses

Continuous variables were characterized as means and 95% CI, categorical variables were characterized by frequencies and percentages. As all participating children with only one parent were included in this study, the analysis in mother-offspring pairs and father-offspring pairs was conducted, respectively. Since the sampling process was a multistage cluster sampling, taking into account the clustered structure of the data, the multilevel mixed-effect model assuming log-binomial distribution was employed to evaluate the association between each paternal or maternal iCVH factor and combined iCVH scores and offspring overweight and obesity. A random intercept was added to count for the clustering of children within schools and provinces. The PR and 95% CI were estimated.

When we have three categories in the outcome, the non-overweight and non-obesity, the overweight, and the obesity, a multinominal logistic regression was applied to further analyze whether there were differences in the association between parental iCVH factors and overweight or obesity, and the relative risk ratio and 95% CI were recorded with the non-overweight and non-obesity as a reference group. Model 1 did not adjust for any variates. Based on the previous knowledge and the characteristics of this database (12, 22), Model 2 was adjusted for parental age, parental educational attainment, residence area, and offspring age, sex, birth weight, fruit consumption, vegetable consumption, SSBs consumption, meat and its product consumption, physical activity, and delivery mode. Since previous studies have found that fathers and mothers may influence boys and girls to different degrees (14), this study further stratified the analysis by sex. Sensitivity analyses were performed by using the WHO growth reference to determine overweight and obesity in children and adolescents (23). All statistical analyses were performed using Stata 15.0 software (StataCorp LLC, TX, USA). All statistical tests were two-sided and were considered statistically significant if *P* < 0.05.

## Results

Characteristics of 9,585 father-offspring pairs and 19,732 mother-offspring pairs are shown in Table 2. In father-offspring pairs, the prevalence of ideal dietary behaviors, ideal BMI status, non-smoking, regular physical activity, and absence of hypertension and diabetes mellitus were 2.7, 63.0, 50.8, 44.0, 95.1, and 98.5%, respectively. The mean iCVH score was 4.5 (SD: 0.9) for fathers and the prevalence of overweight and obesity was 22.0% in children. In the mother-offspring subset, the prevalence of ideal dietary behaviors, ideal BMI status, non-smoking, regular physical activity, absence of hypertension and diabetes mellitus were 3.6, 85.0, 99.0, 52.5, 97.9, and 99.2%, respectively. The mean iCVH score was 5.3 (SD: 0.7) for mothers and the prevalence of overweight and obesity was 23.8% in children.

**Table 2:** Descriptive characteristics of participants enrolled in this study.

**Groups**	**Father-offspring pairs**	**Mother-offspring pairs**
	**(*N* = 9,585)**	**(*N* = 19,732)**
**Parental characteristics**		
Age, year	39.5 (35.8,42.6)	37.3 (33.9,40.4)
BMI, kg/m^2^	24.2 (22.0,26.0)	22.2 (20.1,23.7)
**Educational attainment, %**		
Junior high school or below	3,970 (41.4)	7,507 (38.0)
Senior high school	2,666 (27.8)	5,516 (28.0)
Junior college	1,442 (15.0)	3,612 (18.3)
College or above	1,468 (15.3)	2,998 (15.2)
**Dietary behaviors, %**		
Unideal	9,325 (97.3)	19,021 (96.4)
Ideal	260 (2.7)	711 (3.6)
**BMI status, %**		
Unideal	3,543 (37.0)	2,964 (15.0)
Ideal	6,042 (63.0)	16,768 (85.0)
**Current smoking, %**		
Unideal	4,717 (49.2)	195 (1.0)
Ideal	4,868 (50.8)	19,537 (99.0)
**Physical activity, %**		
Unideal	5,371 (56.0)	9,364 (47.5)
Ideal	4,214 (44.0)	10,368 (52.5)
**Hypertension, %**		
Unideal	469 (4.9)	406 (2.1)
Ideal	9,116 (95.1)	19,326 (97.9)
**Diabetes mellitus, %**		
Unideal	139 (1.5)	149 (0.8)
Ideal	9,446 (98.5)	19,583 (99.2)
**iCVH scores**		
1–3	1,318 (13.8)	197 (1.0)
4	3,370 (35.2)	2,049 (10.4)
5	3,671 (38.3)	9,085 (46.0)
6	1,226 (12.8)	8,401 (42.6)
**Offspring characteristics**		
Age, year	11.2 (8.4, 13.6)	10.9 (8.2, 13.3)
BMI, kg/m^2^	18.5 (15.8, 20.3)	18.5 (15.7, 20.4)
Birth weight, kg	3.3 (3.0, 3.6)	3.3 (3.0, 3.6)
Fruit consumption, serving/week	8.7 (4.0, 12.0)	9.0 (4.0, 14.0)
Vegetable consumption, serving/week	12.3 (7.0, 14.0)	12.5 (7.0, 14.0)
SSBs consumption, serving/week	2.8 (0.0, 3.0)	2.5 (0.0, 3.0)
Meat and its products consumption, serving/week	8.1 (3.0, 9.0)	8.1 (3.0, 9.0)
Physical activity, hours/week	6.7 (1.5, 8.5)	6.0 (1.5, 7.7)
**Sex, %**		
Boys	5,162 (53.9)	9,612 (48.7)
Girls	4,423 (46.1)	10,120 (51.3)
**Residence area, %**		
Urban	5,183 (54.1)	12,443 (63.1)
Rural	4,402 (45.9)	7,289 (36.9)
**Delivery mode, %**		
Natural labor	5,732 (60.7)	10,566 (54.2)
Cesarean section	3,718 (39.3)	8,946 (45.8)
**Nutritional status, %**		
Non-overweight and non-obesity	7,472 (78.0)	15,034 (76.2)
Overweight	1,495 (15.6)	3,230 (16.4)
Obesity	618 (6.4)	1,468 (7.4)

*BMI, body mass index, SSBs, sugar-sweetened beverages, iCVH, ideal cardiovascular health. Ideal dietary behavior was defined as ideal diet score ≥3 components (fruits: ≥3 servings per day; vegetables: ≥4 servings per day; meat and its products: ≤3 servings per week; sugar-sweetened beverages: ≤4 servings per week), ideal BMI status was defined as BMI < 25 kg/m^2^, ideal current smoking status was defined as never smoke, ideal physical activity was defined as ≥150 min/week moderate intensity or ≥75 min/week vigorous intensity or combination, ideal hypertension and diabetes mellitus statuses were defined as the absence of hypertension and diabetes mellitus. One serving of fruit or vegetable is ~120 g. One serving of sugar-sweetened beverage is about 250 ml. One serving of meat is ~100 g. Continuous variables were characterized as means and 95% CI, categorical variables were characterized by frequencies and percentages*.

Table 3 shows the PR of offspring overweight and obesity based on parental individual and combined iCVH factor. In father-offspring pairs, paternal ideal BMI status, non-smoking, absence of hypertension, and absence of diabetes mellitus were identified to correlate with lower prevalence of offspring overweight and obesity, these estimates changed slightly after adjusting for potential confounders. The PR of the paternal individual iCVH factor and offspring prevalence of overweight and obesity ranged from 0.48 (95%CI: 0.42–0.54) to 0.78 (95%CI: 0.69–0.89). While, in mother-offspring pairs, ideal BMI status, regular physical activity, absence of hypertension, and absence of diabetes mellitus were associated with lower prevalence of offspring overweight and obesity, with the PR ranging from 0.50 (95%CI: 0.45–0.56) to 0.85 (95%CI: 0.78–0.93). The prevalence of offspring overweight and obesity decreased with the parental iCVH scores increased (*P* < 0.001). Compared with children whose parents having 1–3 iCVH factors, those whose fathers or mothers adhered to six iCVH factors had 67% (PR: 0.33, 95%CI: 0.25–0.42) and 58% (PR: 0.42, 95%CI: 0.29–0.62) lower prevalence of overweight and obesity, respectively.

**Table 3:** Multilevel mixed-effect models for the association between parental individual and combined iCVH factors and the prevalence of offspring overweight and obesity.

**Group**	***n*/*N* (%)**	**Model 1**		**Model 2**	
		**PR (95% CI)**	***P-*value**	**PR (95% CI)**	***P-*value**
**Father-offspring pairs**
Dietary behaviors
Unideal	2,047/9,325 (22.0)	1 (Reference)		1 (Reference)	
Ideal	66/260 (25.4)	1.14 (0.85, 1.52)	0.393	0.95 (0.64, 1.41)	0.807
BMI status
Unideal	1,083/3,543 (30.6)	1 (Reference)		1 (Reference)	
Ideal	1,030/6,042 (17.0)	0.47 (0.43, 0.53)	<0.001	0.48 (0.42, 0.54)	<0.001
Current smoking
Unideal	1,101/4,717 (23.3)	1 (Reference)		1 (Reference)	
Ideal	1,012/4,868 (20.8)	0.86 (0.78, 0.95)	0.003	0.78 (0.69, 0.89)	<0.001
Physical activity
Unideal	1,198/5,371 (22.3)	1 (Reference)		1 (Reference)	
Ideal	915/4,214 (21.7)	0.95 (0.86, 1.05)	0.315	0.90 (0.80, 1.03)	0.122
Hypertension
Unideal	143/469 (30.5)	1 (Reference)		1 (Reference)	
Ideal	1,970/9,116 (21.6)	0.67 (0.55, 0.83)	<0.001	0.70 (0.54, 0.91)	0.008
Diabetes mellitus
Unideal	43/139 (30.9)	1 (Reference)		1 (Reference)	
Ideal	2,070/9,446 (21.9)	0.68 (0.47, 0.98)	0.039	0.54 (0.34, 0.86)	0.009
iCVH scores		0.73 (0.70, 0.77)	<0.001	0.70 (0.66, 0.75)	<0.001
1–3	419/1,318 (31.8)	1 (Reference)		1 (Reference)	
4	801/3,370 (23.8)	0.69 (0.60, 0.80)	<0.001	0.67 (0.56, 0.80)	<0.001
5	717/3,671 (19.5)	0.54 (0.46, 0.62)	<0.001	0.49 (0.41, 0.58)	<0.001
6	176/1,226 (14.4)	0.36 (0.30, 0.45)	<0.001	0.33 (0.25, 0.42)	<0.001
**Mother-offspring pairs**
Dietary behaviors
Unideal	4,502/19,021 (23.7)	1 (Reference)		1 (Reference)	
Ideal	196/711 (27.6)	1.12 (0.94, 1.33)	0.207	1.04 (0.83, 1.29)	0.745
BMI status
Unideal	1,102/2,964 (37.2)	1 (Reference)		1 (Reference)	
Ideal	3,596/16,768 (21.4)	0.46 (0.42, 0.50)	<0.001	0.50 (0.45, 0.56)	<0.001
Current smoking
Unideal	43/195 (22.1)	1 (Reference)		1 (Reference)	
Ideal	4,655/19,537 (23.8)	1.09 (0.77, 1.55)	0.618	0.90 (0.59, 1.37)	0.615
Physical activity
Unideal	2,332/9,364 (24.9)	1 (Reference)		1 (Reference)	
Ideal	2,366/10,368 (22.8)	0.88 (0.82, 0.94)	<0.001	0.85 (0.78, 0.93)	<0.001
Hypertension
Unideal	118/406 (29.1)	1 (Reference)		1 (Reference)	
Ideal	4,580/19,326 (23.7)	0.72 (0.58, 0.90)	0.004	0.64 (0.49, 0.84)	0.002
Diabetes mellitus
Unideal	48/149 (32.2)	1 (Reference)		1 (Reference)	
Ideal	4,650/19,583 (23.7)	0.63 (0.44, 0.90)	0.011	0.60 (0.38, 0.95)	0.028
iCVH scores		0.72 (0.69, 0.76)	<0.001	0.73 (0.68, 0.77)	<0.001
1–3	77/197 (39.1)	1 (Reference)		1 (Reference)	
4	682/2,049 (33.3)	0.77 (0.56, 1.04)	0.092	0.84 (0.57, 1.24)	0.379
5	2,237/9,085 (24.6)	0.50 (0.37, 0.68)	<0.001	0.56 (0.39, 0.82)	0.003
6	1,702/8,401 (20.3)	0.39 (0.29, 0.52)	<0.001	0.42 (0.29, 0.62)	<0.001

*BMI, body mass index; iCVH, ideal cardiovascular health. Ideal dietary behavior was defined as ideal diet score ≥3 components (fruits: ≥3 servings per day; vegetables: ≥4 servings per day; meat and its products: ≤3 servings per week; sugar-sweetened beverages: ≤4 servings per week), ideal BMI status was defined as BMI < 25 kg/m^2^, ideal current smoking status was defined as never smoke, ideal physical activity was defined as ≥150 min/week moderate intensity or ≥75 min/week vigorous intensity or combination, ideal hypertension and diabetes mellitus statuses were defined as the absence of hypertension and diabetes mellitus. One serving of fruit or vegetable is ~120 g. One serving of sugar-sweetened beverage is about 250 ml. One serving of meat is ~100 g. Model 1 did not adjust for any variates. Model 2 was adjusted for parental age, parental educational attainment, residence area, and offspring age, sex, birth weight, fruit consumption, vegetable consumption, SSBs consumption, meat and its products consumption, physical activity, and delivery mode. Model goodness-of-fit test: P < 0.01*.

The associations between parental iCVH factors and the prevalence of overweight and obesity among offspring were almost consistent, although the PRs were smaller in obese children than in overweight children (Supplementary Table 1). In sensitivity analysis, the results were essentially similar when the outcome was determined by different criteria (Supplementary Table 2). Furthermore, stratified by sex, the relationships between iCVH factors of fathers and mothers and prevalence of overweight and obesity in boys and girls were similar, with a stronger intergenerational association was observed in mother-daughter pairs (Table 4).

**Table 4:** Adjusted multilevel mixed-effect models for the association between parental iCVH factors and the prevalence of offspring overweight and obesity, stratified analysis by sex.

**Parental iCVH factors**	**Boys**		**Girls**	
	**PR (95% CI)**	***P-*value**	**PR (95% CI)**	***P*-value**
**Father-offspring pairs**
Ideal dietary behaviors	0.95 (0.56, 1.60)	0.842	0.91 (0.50, 1.66)	0.761
Ideal BMI status	0.49 (0.41, 0.57)	<0.001	0.46 (0.38, 0.56)	<0.001
Ideal current smoking	0.77 (0.66, 0.91)	0.002	0.78 (0.64, 0.95)	0.013
Ideal physical activity	0.85 (0.72, 1.01)	0.06	0.97 (0.79, 1.18)	0.736
Ideal hypertension	0.63 (0.44, 0.90)	0.01	0.77 (0.51, 1.15)	0.196
Ideal diabetes mellitus	0.63 (0.36, 1.11)	0.111	0.39 (0.18, 0.84)	0.016
iCVH scores	0.69 (0.63, 0.76)	<0.001	0.71 (0.63, 0.79)	<0.001
1–3	1 (Reference)		1 (Reference)	
4	0.63 (0.50, 0.80)	<0.001	0.72 (0.54, 0.95)	0.019
5	0.48 (0.38, 0.60)	<0.001	0.47 (0.36, 0.63)	<0.001
6	0.31 (0.22, 0.43)	<0.001	0.34 (0.23, 0.52)	<0.001
**Mother-offspring pairs**
Ideal dietary behaviors	1.25 (0.94, 1.66)	0.124	0.80 (0.56, 1.15)	0.226
Ideal BMI status	0.53 (0.45, 0.61)	<0.001	0.47 (0.40, 0.55)	<0.001
Ideal current smoking	0.91 (0.51, 1.62)	0.747	0.93 (0.51, 1.71)	0.818
Ideal physical activity	0.81 (0.72, 0.91)	<0.001	0.90 (0.79, 1.02)	0.107
Ideal hypertension	0.66 (0.45, 0.98)	0.038	0.62 (0.42, 0.91)	0.015
Ideal diabetes mellitus	0.64 (0.34, 1.24)	0.186	0.55 (0.29, 1.04)	0.067
iCVH scores	0.71 (0.66, 0.78)	<0.001	0.74 (0.68, 0.81)	<0.001
1–3	1 (Reference)		1 (Reference)	
4	0.99 (0.57, 1.70)	0.962	0.72 (0.42, 1.24)	0.231
5	0.62 (0.36, 1.04)	0.072	0.52 (0.31, 0.89)	0.016
6	0.46 (0.27, 0.78)	0.004	0.39 (0.23, 0.67)	0.001

*BMI, body mass index; iCVH, ideal cardiovascular health. Ideal dietary behavior was defined as ideal diet score ≥3 components (fruits: ≥3 servings per day; vegetables: ≥4 servings per day; meat and its products: ≤3 servings per week; sugar-sweetened beverages: ≤4 servings per week), ideal BMI status was defined as BMI < 25 kg/m^2^, ideal current smoking status was defined as never smoke, ideal physical activity was defined as ≥150 min/week moderate intensity or ≥75 min/week vigorous intensity or combination, ideal hypertension and diabetes mellitus statuses were defined as the absence of hypertension and diabetes mellitus. One serving of fruit or vegetable is ~120 g. One serving of sugar-sweetened beverage is about 250 ml. One serving of meat is ~100 g. The model was adjusted for parental age, parental educational attainment, residence area, and offspring age, birth weight, fruit consumption, vegetable consumption, SSBs consumption, meat and its products consumption, physical activity, and delivery mode. Model goodness-of-fit test: P < 0.01*.

## Discussion

In this cross-sectional study with a large national sample, we assessed the associations between parental individual, and combined, cardiovascular health factors and the PR of overweight and obesity in their offspring. Findings suggested that parental adherence to an iCVH status was associated with a lower prevalence of overweight and obesity in their offspring, and the more iCVH factors parents satisfied with, the lower prevalence of overweight and obesity in their offspring. In addition, we also found that both paternal and maternal cardiovascular health statuses were very important in reducing the prevalence of offspring overweight and obesity.

With the rapid increase of overweight and obesity among the young population, it is important to identify potential correlated factors. In this study, we found a lower prevalence of overweight and obesity in children whose parents were healthy in BMI, absence of hypertension, and absence of diabetes mellitus, our findings were in line with several previous studies in other populations, which demonstrated family aggregation of obesity and related phenotypes. For example, Whitaker et al. (24) found that parental obesity can be used to predict obesity in their offspring. Yoo and Park (9) found a positive correlation between parental hypertension and the risk of overweight in offspring. Besides, a cohort study from China also demonstrated that maternal history of diabetes could lead to a higher risk of obesity in offspring (25). Previous studies reported that possible pathways of those associations could be attributed to both genetics and lifestyle behaviors from parents to offspring (26–29).

Currently, evidence regarding the association between parental overall cardiovascular health and offspring overweight and obesity is limited. A cohort study (12) consisted of 16,945 mothers and 24,289 children had demonstrated that mothers who adhered to a combined healthy lifestyle (characterized by healthy BMI, high-quality diet, regular exercise, non-smoking, and light to moderate alcohol intake) were associated with a 75% lower risk of obesity in their offspring. Another cohort study also found that adherence of women to an overall healthy lifestyle before pregnancy was strongly correlated with a lower risk of pediatric obesity in offspring (30), the results from those studies were consistent with us. One possible explanation of this correlation was the same living environment shared by parents and their offspring could lead to a similar selection of lifestyle behaviors. Parents could set a good example for their offspring, as the children are good at imitation and can easily develop behaviors or cognitive functions by observing and learning from their parents (31).

Familial aggregation to obesity and intergenerational transmission had been found (32), but rarely had considered the effects of father-offspring pairs and mother-offspring pairs simultaneously. Although some studies had shown that mothers had a greater influence on their offspring than fathers (26), this study was in line with the study conducted in India (33), which found that paternal and maternal cardiovascular health statuses had a similar impact on the risk of offspring overweight and obesity. This emphasized that both fathers and mothers play an important role in the development of offspring obesity. Unlike previous studies that have found the association between generations of the same sex was greater (14), our results showed that the associations between fathers and mothers iCVH status and offspring overweight and obesity were similar, although the link was a little greater in mother-daughter. This suggested that intervention strategies prevent childhood obesity need to consider the indispensable role of both father and mother involvement in both boys and girls.

This study has several strengths. Based on a national multicenter sample, this study has a large enough sample to analyze the relationship between parental iCVH factors and childhood overweight and obesity. In addition, this study is based on the modifiable parental iCVH factors, covering both fathers and mothers, focusing on combined factors, and the results have important indicating significance for the early comprehensive intervention of childhood obesity. It is suggested that the intervention of a healthy lifestyle for parents and promotion of cardiovascular health could be a potentially effective measure to prevent overweight and obesity in their offspring. However, this study also had several limitations. First, the cross-sectional design is prone to reverse causation and does not infer the temporal order of the relationship. Second, for each child, we have only collected cardiovascular health information for one parent (either father or mother), thus the correlations were assessed in two separate subsets, which may limit the comparability between fathers and mothers. Moreover, the effect size of the association was larger in the father-offspring pairs, but the sample size of fathers to fill out the questionnaire was smaller, so the effect may be underestimated. Third, parental cardiovascular health factors were collected from a questionnaire, rather than objective measurements. There may be recall bias or overestimation of cardiovascular health factors. Future studies would be preferable to have the information verified by medical records, registries, or objective measurements to minimize recall bias and reporting bias. Fourth, although we adjusted for the potential confounding factors, residual confounding and unknown factors could not be totally ruled out. Fifth, we used a modified definition of the ideal diet to comply with Chinese dietary habits based on the questionnaires in the current survey. Thus, our results could not be compared directly with other studies conducted in the Western population using the iCVH criteria of the AHA. Finally, although this study is a multicenter sampling method, the subjects were all Chinese Han population, it is necessary to be cautious when the results were extrapolated, especially in some regions with unique dietary habits and cultural characteristics.

## Conclusion

In conclusion, our findings suggested that parental adherence to an iCVH status was associated with a substantially lower prevalence of childhood overweight and obesity in their offspring. Our findings highlighted the critical role of parental cardiovascular health status on overweight and obesity in offspring and supported the intervention strategy that parents should involve in the obesity intervention program for children.

## Data Availability Statement

The data supporting the conclusions of this article will be made available from the corresponding author upon request.

## Ethics Statement

The studies involving human participants were reviewed and approved by the Ethical Committee of Peking University (IRB No. 0000105213034). Written informed consent to participate in this study was provided by the participants' legal guardian/next of kin.

## Author Contributions

YL and DG carried out the data analysis, drafted the initial manuscript, and revised the manuscript. YL and ZY designed the study and performed the data analysis. YM and MC interpreted the results and reviewed the manuscript. JM contacted the scene, critically reviewed the manuscript, and contributed to the interpretation of results. YD and BD participated in reviewing and revising the manuscript. All authors read and approved the final manuscript.

## Funding

This study was granted by the Research Special Fund for Public Welfare Industry of Health (201202010) and the China Post-doctoral Science Foundation (BX20200019 and 2020M680266).

## Conflict of Interest

The authors declare that the research was conducted in the absence of any commercial or financial relationships that could be construed as a potential conflict of interest.

## Publisher's Note

All claims expressed in this article are solely those of the authors and do not necessarily represent those of their affiliated organizations, or those of the publisher, the editors and the reviewers. Any product that may be evaluated in this article, or claim that may be made by its manufacturer, is not guaranteed or endorsed by the publisher.
