# Supplementary material for: Parental Adherence to Ideal Cardiovascular Health Status Was Associated With a Substantially Lower Prevalence of Overweight and Obesity in Their Offspring Aged 6–18 Years
Source: Front Nutr. 2021 Sep 20;8:715171. doi: 10.3389/fnut.2021.715171 (PMC8488122; doi:10.3389/fnut.2021.715171)
Supplement: Supplementary file 1 [file Table_1.pdf]

Supplementary Table 1 Adjusted Multinominal logistic regression for association between parental iCVH factors and the prevalence of offspring overweight and obesity

Parental iCVH factors	Overweight		Obesity	
	RRR (95% CI)	*P* value	RRR (95% CI)	*P* value
**Father-offspring pairs**				
Ideal Dietary behaviors	0.98(0.63,1.54)	0.939	1.16(0.62,2.18)	0.633
Ideal BMI status	0.53(0.46,0.61)	<0.001	0.34(0.27,0.42)	<0.001
Ideal Current smoking	0.82(0.72,0.95)	0.006	0.65(0.53,0.81)	<0.001
Ideal Physical activity	0.91(0.79,1.05)	0.201	0.91(0.73,1.13)	0.401
Ideal Hypertension	0.70(0.52,0.94)	0.019	0.54(0.36,0.82)	0.003
Ideal Diabetes mellitus	0.49(0.30,0.80)	0.004	0.60(0.28,1.30)	0.195
iCVH scores	0.73(0.68,0.79)	<0.001	0.59(0.53,0.67)	<0.001
1-3	1 [Reference]		1 [Reference]	
4	0.70(0.57,0.86)	0.001	0.55(0.42,0.72)	<0.001
5	0.53(0.44,0.66)	<0.001	0.34(0.25,0.46)	<0.001
6	0.37(0.28,0.49)	<0.001	0.20(0.12,0.32)	<0.001
**Mother-offspring pairs**				
Ideal Dietary behaviors	1.05(0.81,1.34)	0.726	1.27(0.91,1.76)	0.158
Ideal BMI status	0.56(0.49,0.63)	<0.001	0.37(0.31,0.44)	<0.001
Ideal Current smoking	1.04(0.63,1.72)	0.876	0.65(0.36,1.19)	0.162
Ideal Physical activity	0.86(0.78,0.94)	0.002	0.83(0.73,0.95)	0.009
Ideal Hypertension	0.68(0.50,0.93)	0.015	0.58(0.38,0.87)	0.009
Ideal Diabetes mellitus	0.67(0.40,1.13)	0.132	0.51(0.26,0.99)	0.047
iCVH scores	0.76(0.71,0.82)	<0.001	0.63(0.57,0.69)	<0.001
1-3	1 [Reference]		1 [Reference]	
4	0.78(0.51,1.19)	0.243	0.99(0.55,1.78)	0.967
5	0.56(0.37,0.85)	0.006	0.56(0.31,0.99)	0.046
6	0.44(0.29,0.67)	<0.001	0.36(0.20,0.64)	0.001

BMI: body mass index, iCVH: ideal cardiovascular health. Ideal dietary behavior was defined as ideal diet score ≥3 components (Fruits: ≥3 servings per day; Vegetables: ≥4 servings per day; Meat and its products: ≤3 servings per week; Sugar-sweetened beverages: ≤4 servings per week), ideal BMI status was defined as BMI <25kg/m2, ideal current smoking status was defined as never smoke, ideal physical activity was defined as ≥150min/week moderate intensity or ≥75 min/week vigorous intensity or combination, ideal hypertension and diabetes mellitus status was defined as absence of hypertension and diabetes mellitus. One serving of fruit or vegetable is approximately 120 g. One serving of sugar-sweetened beverage is about 250 ml. One serving of meat is approximately 100 g. Model was adjusted for parental age, parental educational attainment, residence area, and offspring age, sex, birth weight, fruit consumption, vegetable consumption, SSBs consumption, meat and its products consumption, physical activity, delivery mode. Model goodness of fit test: $P<0.01$.

Supplementary Table 2 Adjusted multilevel mixed effect models for association between parental iCVH factors and the prevalence of offspring overweight and obesity (as determined by WHO)

Parental iCVH factors	Father-offspring pairs		Mother-offspring pairs	
	PR (95% CI)	*P* value	PR (95% CI)	*P* value
Ideal Dietary behaviors	1.16(0.81,1.68)	0.412	1.03(0.83,1.27)	0.812
Ideal BMI status	0.51(0.46,0.58)	<0.001	0.49(0.44,0.54)	<0.001
Ideal Current smoking	0.83(0.74,0.93)	0.002	0.81(0.54,1.21)	0.308
Ideal Physical activity	0.96(0.85,1.08)	0.48	0.84(0.78,0.92)	<0.001
Ideal Hypertension	0.75(0.58,0.97)	0.03	0.72(0.55,0.95)	0.021
Ideal Diabetes mellitus	0.49(0.31,0.76)	0.002	0.57(0.36,0.89)	0.013
iCVH scores	0.74(0.69,0.79)	<0.001	0.72(0.68,0.77)	<0.001
1-3	1 [Reference]		1 [Reference]	
4	0.70(0.59,0.83)	<0.001	0.88(0.60,1.29)	0.523
5	0.54(0.45,0.64)	<0.001	0.58(0.40,0.84)	0.004
6	0.39(0.31,0.49)	<0.001	0.43(0.30,0.63)	<0.001

BMI: body mass index, iCVH: ideal cardiovascular health. Ideal dietary behavior was defined as ideal diet score ≥3 components (Fruits: ≥3 servings per day; Vegetables: ≥4 servings per day; Meat and its products: ≤3 servings per week; Sugar-sweetened beverages: ≤4 servings per week), ideal BMI status was defined as BMI <25kg/m2, ideal current smoking status was defined as never smoke, ideal physical activity was defined as ≥150min/week moderate intensity or ≥75 min/week vigorous intensity or combination, ideal hypertension and diabetes mellitus status was defined as absence of hypertension and diabetes mellitus. One serving of fruit or vegetable is approximately 120 g. One serving of sugar-sweetened beverage is about 250 ml. One serving of meat is approximately 100 g. Model was adjusted for parental age, parental educational attainment, residence area, and offspring age, sex, birth weight, fruit consumption, vegetable consumption, SSBs consumption, meat and its products consumption, physical activity, delivery mode. Model goodness of fit test: $P<0.01$.
